# Supplementary material for: New Canadian and Provincial Records of Coleoptera Resulting from Annual Canadian Food Inspection Agency Surveillance for Detection of Non-Native, Potentially Invasive Forest Insects
Source: Insects. 2022 Aug 6;13(8):708. doi: 10.3390/insects13080708 (PMC9408973; doi:10.3390/insects13080708)
Supplement: Supplementary file 1 [file insects-13-00708-s001.zip › insects-1808477-supplementary.pdf]

Supplementary Materials

Table S1. Traps and lures used by the Canadian Food Inspection Agency and the years they were deployed in annual surveys for adventive bark- and wood-boring insects inadvertently introduced to Canada via international import of goods, human-assisted movement, or natural dispersal. With exception of monochamol and ipsenol (released from the same lure), each component of a multi-lure combination consisted of a separate lure and each lure was placed on the same trap, e.g., traps baited with the 'general longhorn lure' had three lures attached: fuscumol, fuscumol acetate, and UHR ethanol UHR = Ultra High Release rate.orrected

**Trap**	**Lure**	**Years deployed**	**Target taxa**	**New Provincial Records**[3]
Black Lindgren, collecting cup 30 cm above ground	UHR ethanol	2011	Curculionidae, (esp. ambrosia beetles), Cerambycidae that attack broadleaf trees	1
	UHR ethanol + UHR alpha-pinene	2011	Cerambycidae, Buprestidae, Curculionidae that attack conifers	1
	Racemic 3-hydroxyhexan-2-one + racemic 3-hydroxyoctan-2-one + UHR ethanol	2012–16	Cerambycidae (esp. Cerambycinae), Curculionidae, that attack either conifers or broadleaf trees	2
	(*E*,*Z*)-fuscumol + (*E*,*Z*)-fuscumol acetate + UHR ethanol (= general longhorn lure)	2015–21	Cerambycidae (esp. Lamiinae, Spondylidinae), Curculionidae that attack either conifers or broadleaf trees	14c
	Monochamol + ipsenol + UHR α-pinene + UHR ethanol	2015–21	Cerambycidae (esp. Lamiinae), Curculionidae that attack conifers	8
	3-methyl-2-buten-1-ol[1]	2013		1
Green Lindgren, placed in tree canopy[2]	Racemic 3-hydroxyhexan-2-one + racemic 3-hydroxyoctan-2-one + UHR ethanol	2016	Cerambycidae, Buprestidae, Curculionidae	1

			more active in tree canopy	
Black Lindgren, placed in canopy[2]	(*E*,*Z*)-fuscumol + (*E*,*Z*)-fuscumol acetate + UHR ethanol	2018–19	Cerambycidae, Curculionidae more active in tree canopy	1
Green Lindgren, placed in tree canopy[2]	(*E*,*Z*)-fuscumol + (*E*,*Z*)-fuscumol acetate + UHR ethanol	2018–21	Cerambycidae, Buprestidae, Curculionidae more active in tree canopy	2

[1] Ontario Ministry of Natural Resources record; [2] Canopy traps were deployed in only a small number of survey locations as part of pilot project [3] Number of new species records detected by the trap-lure combination; this sums to more than a total of 31 records because some species were detected by more than one type of trap-lure combination.

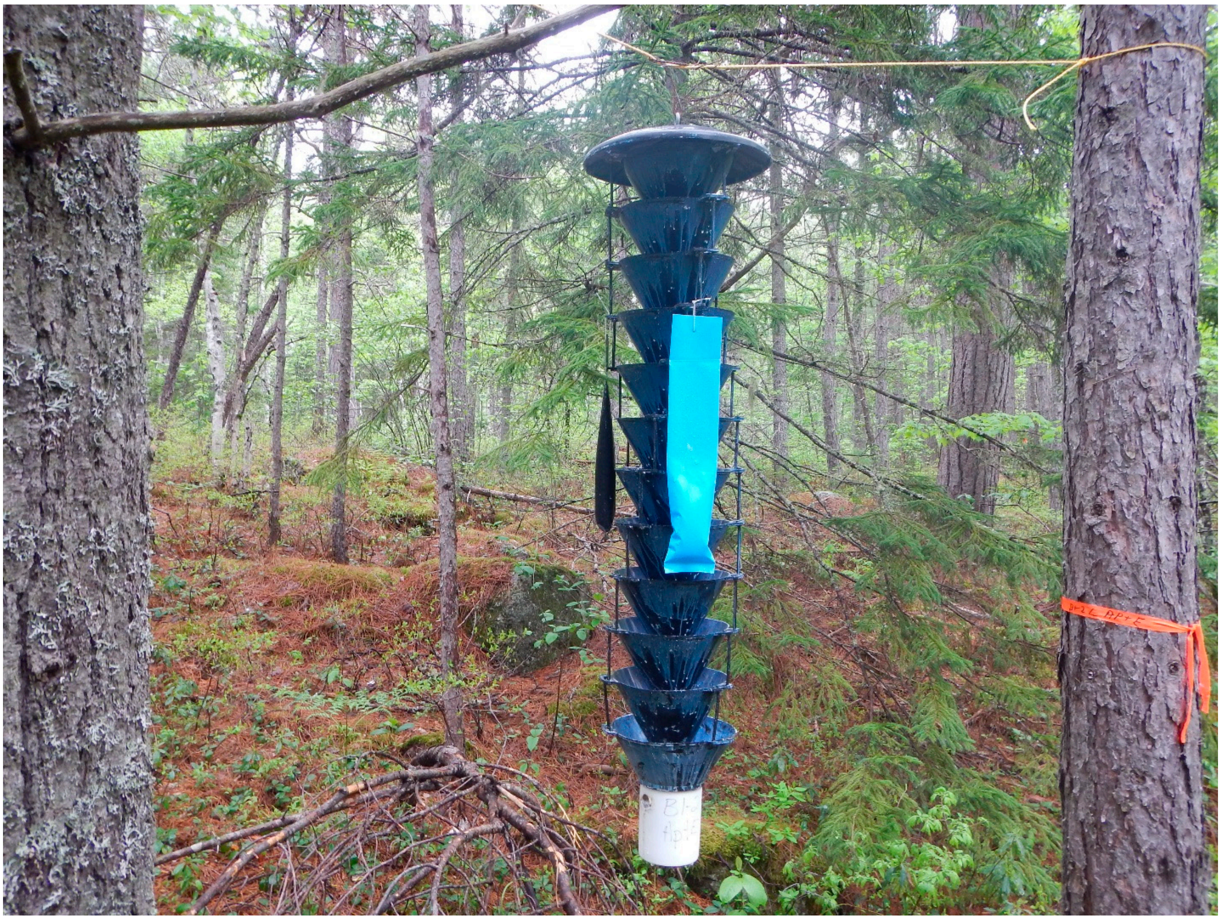

Figure S1. Black 12-funnel Lindgren trap suspended from rope tied between trees with collecting cup 30-50 cm above forest floor.

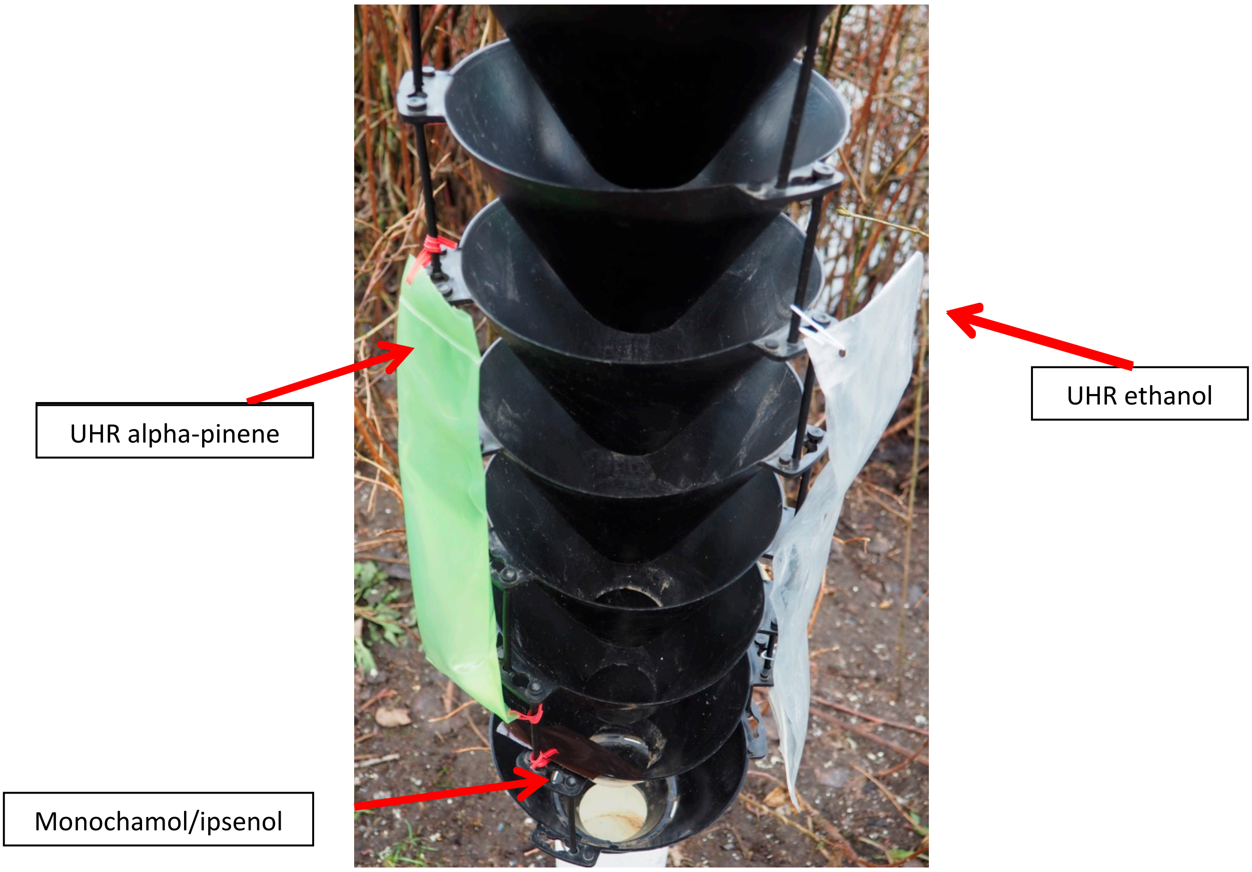

Figure S2. Close up of black Lindgren funnel trap showing example of lure placement for traps baited with monochamol/ipsenol, UHR ethanol and UHR alpha-pinene.

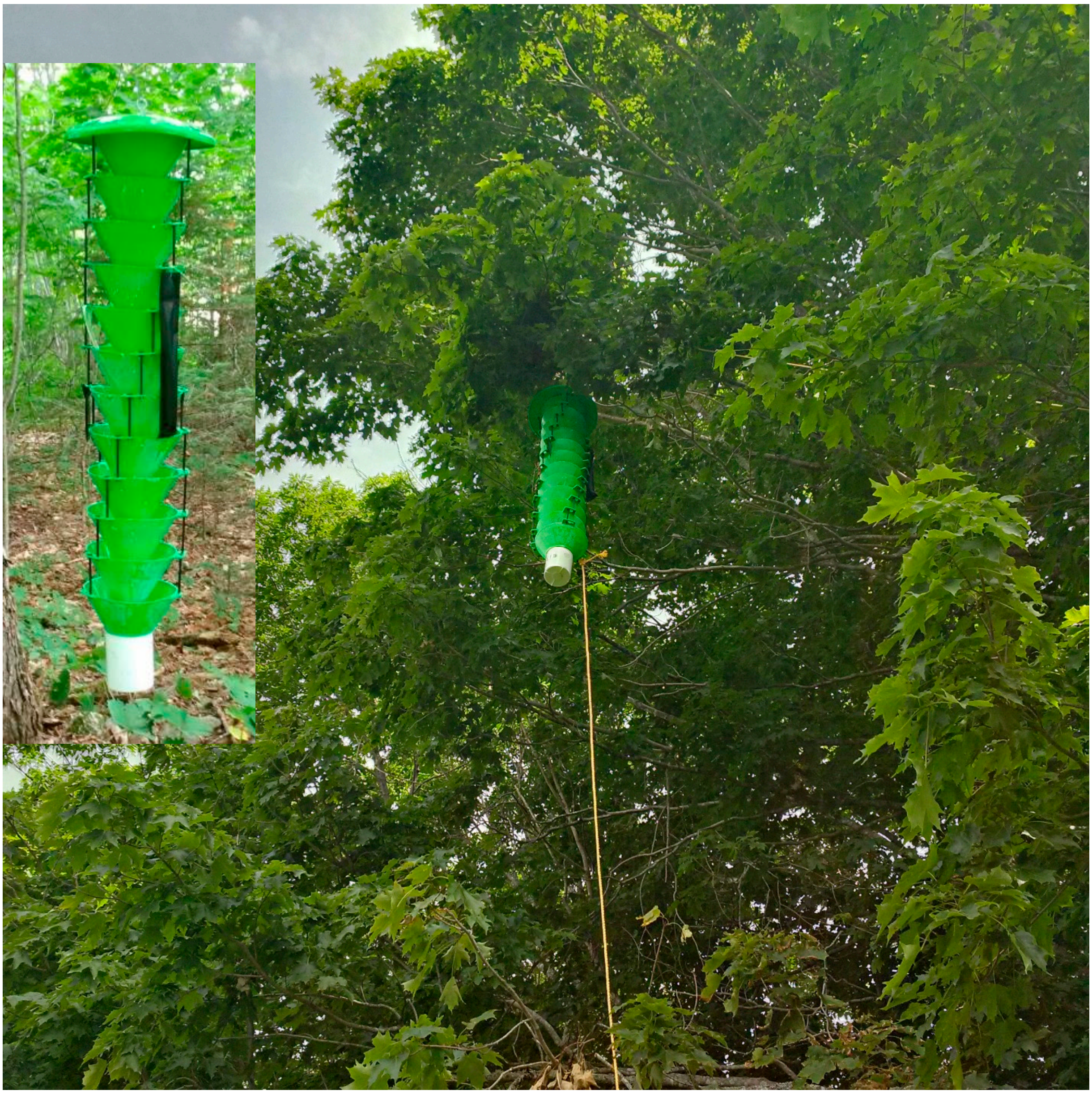

Figure S3. Green 12-funnel Lindgren trap (inset) and showing trap position in the upper canopy of a broadleaf tree.
